# Cross-Cultural Differences in Sexting Practices between American and Spanish University Students

**DOI:** 10.3390/ijerph18042058

**Published:** 2021-02-20

**Authors:** Aina M. Gassó, José R. Agustina, Esperanza Goméz-Durán

**Affiliations:** 1 Faculty of Law, Universitat Internacional de Catalunya, 08017 Barcelona, Spain; 2 Faculty of Law, Universitat Abat Oliba CEU, 08022 Barcelona, Spain; jagustinas@uao.es; 3 School of Medicine, Universitat Internacional de Catalunya, 08017 Barcelona, Spain; elgomez@uic.es

**Keywords:** sexting, online sexual victimization, cultural differences, cross-culture

## Abstract

Despite the growing body of research regarding sexting and online sexual victimization, there is little evidence exploring cultural differences in association with those behaviors. The aim of this study was to examine cultural differences in sexting practices by comparing an American sample and a Spanish sample of university students. The original sample was composed of 1799 college students, including 1386 Spanish college students and 413 American Students, with 74% of female participants, and ages ranging from 18 to 64 years old (mean age = 21.26, SD= 4.61). Results indicate that American students sext more than Spanish students and have higher probabilities of being victims of nonconsensual dissemination of their sexual content. However, Spanish students receive more sexts than American students. Although our results show differences between the Spanish and the American samples that might be modulated by cultural factors, the vulnerability of females regarding sexting remains unchanged. Additionally, differences in specific characteristics of the behaviors (such as perceived risk, receiver of the sexual content, intensity of the sexual content, and motive for sexting) were also studied. Further results and implications are discussed in relation to cultural differences.

## 1. Introduction

Research on sexting has been increasingly growing over the past few years. According to Agustina [1], the exchange of sexual messages has existed throughout history, however, new technologies have facilitated the exchange of images and videos, which are undoubtedly more explicit and might have a stronger impact both on the sender and on the receiver. This exchange of erotic or sexual content has become to be known as sexting, a term first used in 2005 by the Sunday Telegraph, which unified the terms “sex” and “texting”, and tried to describe a new and incipient phenomenon where people were exchanging text messages with erotic or sexual content. The term sexting has evolved over the years, and despite the fact that there is still no clear consensus around its definition sexting is understood by many authors as creating, sending, forwarding and/or receiving nude or sexually explicit images or videos through any electronic device [2,3]. 

Up to date literature indicates that sexting increases with age, and that it is most frequent between 18 and 35 years old [4,5,6]. The literature review carried out by Klettke et al. [7] shows that the estimated mean prevalence regarding the engagement in general sexting behaviors is 53.3%, while studies looking specifically into the sending of sexts with sexual photos showed an estimated mean prevalence of 48.6%, with slightly higher rates for receiving sexts, 56.6%. Studies examining prevalence rates amongst university students report similar findings. Benotsch et al. [8] surveyed 763 undergraduate students and their results indicated that 44% of the sample had either sent or received a sext, while Dir et al. [9] reported that 46.6% had sent a sexual picture and 64.2% had received a sexual picture. Other studies carried out during the same time period and using undergraduate samples found similar results [10,11,12].

Studies examining sexting prevalence and characteristics in Spanish adult population have reported that 66.8% of the participants were involved in at least one type of sexting, and 46.7% of participants had been involved in sexting three or more times [4]. A recent study examining sexting in Spanish university students found slightly lower prevalence rates, reporting that 37% of participants had sent sexual content of themselves and 60% of participants had received sexts [13]. Additionally, Spanish data indicates that around 1% of adult population and, more specifically, 3% of university students, have been victims of nonconsensual dissemination of sexting [4,13]. Studies examining sexting data from the United States of America in adult population indicate similar sexting rates, with most research reporting prevalence rates between 45% and 50% for sending sexts, and around 60% for receiving sexts [9,10,11].

To date, literature has examined different sexting-related issues such as perceived risk and upsetness generated by the behavior, characteristics of the sender and receiver of the sexts, sexual intensity of the shared content, and motive for engaging in the behavior [7,14,15]. 

Regarding perceived risk when engaging in sexting, Kopecký [16] found that 73% of participants in their study thought sexting was risky and dangerous, whilst results from the NCPTUP [14] indicated that 75% of teens and 71% of young adults believed that sending sexts with sexually suggestive content could have serious negative consequences. Additionally, a recent study carried out by Villacampa [6] found that 44% of the sample felt upset by the engagement in sexting behaviors, and when asked about nonconsensual dissemination of sexting, the majority of participants reported negative feelings. Similarly, results from the AP-MTV survey [15] reported that 53% of participants found the experience of sexting deeply upsetting and Dir and Cyders [17] in a sample of US university students found that those who perceive higher risk in sexting are expected to engage less in the behavior. 

Moreover, studies examining sender/receiver of the sexts reported that participants indicated they usually engaged in sexting with their friends (12%) or with their romantic partners (10%), whilst results from the AP-MTV survey [15] reported that 10% of participants sexted with a stranger. With regards to the sexual intensity of the shared content, Villacampa [6] found that 65% of sexting sexual content shared by the participants in her study was clearly sexual and highly pornographical, whilst in 35% of the cases, images were not clearly sexual with 29% of participants reporting that images contained people in underwear (moderately sexual) and 3% indicating images which were only sexually suggestive (subtle sexual content).

Studies analyzing why people engage in sexting behaviors have shown homogeneous results. Henderson and Morgan [10] analyzed the reasons why people sext, finding that ‘to be sexy or initiate sexual activity’, was the most highly reported reason (85% of the sample), followed by ‘gaining attention from a partner’ (80%), ‘for fun’ (65%), and ‘pressure from friends or dating partners’ (30%) [7]. Similarly, Kopecký [16] found the most frequently reported motives for sexting to be boredom, to initiate sexual activity, under peer pressure, accidentally or to gain sexual attention from the recipient. Drouin et al. [18] reported the most frequent motivations for sexting amongst college students varied depending on the relationship status, but were mainly sexual motivations (flirting, answering a partner request and to initiate sexual activity). Results from the NCTPUP [14] showed that the three primary reasons young adults (ages 20–26) gave for sexting were to be ‘fun or flirtatious’ (72% women and 70% men), ‘as a sexy present for their boyfriend’ (59% of women), and in response to receiving similar content (41% of women and 51% of men), and, Villacampa [6] found that 34% of the sample reported sexting as a ‘joke’, 29% did not know why they engaged in sexting, and 23% in the context of intimate partner relationships. Regarding the nonconsensual dissemination of sexts, the AP-MTV survey [15] found that 17% of those who reported receiving sexts had passed them along to someone else, with 55% sharing the sext with more than one person. The most common reasons for forwarding sexts included assuming that others would want to see them (55%), ‘showing off’ (35%), as a joke (31%), to be funny (30%), and boredom (26%).

Nevertheless, cross-cultural studies report contradictory data about sexting behaviors. The sexual culture of human beings is strongly affected by societal influences [19]. As a result, social structures can facilitate or discourage certain sexual activities, and this, in turn, can also affect attitudes toward sexual behaviors [19]. It has been previously suggested that an alignment with more conservative politics is related to less supportive attitudes towards sexual behaviors [19]. Specifically regarding sexting, the study carried out by EU Kids Online has shown that culture might play an important role in how people engage in sexting, by showing differences across different cultures [20]. For instance, Italy and Spain which are culturally similar countries reported different prevalence rates: 30% of Spanish participants reported receiving sexts and 9% sending sexts, whilst 9% of Italian participants reported receiving sexts and 2% sending sexts. On the other hand, countries which could be thought of being culturally different, such as Spain and Norway reported more similar findings with 30% of Norwegian participants reporting having received sexts and 7% having sent sexts. Nevertheless, the cross-cultural study in 10 countries carried out by Morelli et al. [21] reported remarkably higher percentages in Italy: 47.4% of Italian participants reported sending their own sexts; similarly to participants from Belgium (47.8%), Czech Republic (45.9%) and Russia (45.7%); with a higher frequency than participants from Poland (40.6%), Turkey (30.6%), Ireland (28.4%), Uganda (16.9%), or China (14.3%). American participants reported the highest frequency of sending their own sexts (54.4%). Spain was not involved in this study. Furthermore, a recent cross-cultural study examining sexting differences between Spain and Colombia found that sexting was strongly influenced by the country and highlighted that sociocultural aspects and gender constitute differential variables that should be studied [22]. 

Regarding the present study, Spain and the USA are comparable in the use of the internet among young people [23]. However, they differ in problematic internet use rate with data suggesting that 36% of American university students show online problematic experiences [24] whilst only 10% of Spanish university students reported online problematic experiences [25]. Considering the stated disparate historical, cultural and socioeconomic factors that influence sexual behavior, there is much reason to believe that American and Spaniards will differ in sexting behavior engagement. The difficulty in comparing results from individual studies in different countries due to the use of different measures of sexting has been highlighted [26] and the few cross-cultural studies published so far do not discuss cultural differences across countries. Therefore, the present study aims to fill this gap in the literature, allowing for a deeper understanding of what role cultural differences might play in sexting engagement. Thus, the aim of this paper is to examine and explore cultural differences in sexting practices by comparing an American sample and a Spanish sample of university students. We hypothesize that American and Spanish university students will engage differently in all of the measured sexting behaviors, due to cultural differences.

## 2. Materials and Methods

### 2.1. Participants

The original sample consisted of 1799 college students, including 1386 Spanish college students (77%) and 413 American college students (23%). Out of the total sample, 1311 were women (74%) and 460 were men (26%), with ages ranging from 18 to 64 years old (mean age = 21.26, SD = 4.61). The descriptive statistics for the total sample can be found in Table 1. 

### 2.2. Instruments

#### 2.2.1. Sexting Questionnaire

For the purpose of this research, we defined sexting as creating and sending or receiving nude or sexually explicit images or videos through any electronic device (i.e., excluding text messages) [2]. 

We used a modified version of the JOV-Q [27] to assess three different sexting behaviors: primary sexting (creating and sending sexts), secondary sexting (receiving sexts) and being a victim of nonconsensual dissemination of sexts (NCDS) [6]. For each of the measured behaviors, we asked how many times they engaged in the behavior, on a 6-point Likert scale (0 = never; 6 = everyday). This then was recoded as lifetime prevalence (Yes, at least once/ No, never engaged in this behavior). We also asked how old they were when they first engaged in the behavior, the degree of sexual intensity of the pictures involved in the behavior (0 = very subtle, 10 = clearly sexual) and the motive for engaging in the behavior. For primary sexting, we asked participants to assess how risky they thought the behavior was (0 = not risky; 10 = very risky), and to whom they had sent the sexual content. For secondary sexting and being a victim of NCDS, we asked participants to assess how upset they felt about the behavior (0 = not upset; 10 = very upset), and who had sent them/done to them the behavior. 

#### 2.2.2. Sociodemographic Questionnaire

We included questions about age, sex, marital status, parental marital status, place of residence, employment situation, academic situation, and questions about frequency and use of phones and social media. 

### 2.3. Procedure

The study was approved by the Ethics Committee of the International University of Catalunya (UIC Barcelona), with ethical approval code DRET-2018–02, and by the Ethics Committee of the Texas State University, with ethical approval code IRB-6252. Participation was voluntary and responses were anonymous to promote openness and honesty. The survey was administered online using Qualtrics online survey platform. To gather the Spanish sample, the survey link was sent to university professors from Spanish universities with a request to pass it on to their students. To gather the American sample, the survey link was shared with students from Texas State University. The participating students then self-selected to take part in their own time. The questionnaire took approximately 15–20 min to complete, and once completed, students were given information on community resources in case of distress and the email address to contact the investigators in case of concerns. No participant contacted the investigators. 

## 3. Results

### 3.1. Sample Demographic Characteristic

Table 1 shows the descriptive statistics of demographic and background variables for the total sample, and by nationality. Out of the total sample (*n* = 1799), 55.0% were single and 93.1% were undergraduate students. The greater majority of participants owned a smartphone (98.3%), 97% used social media, and almost half of the sample used the internet more than 3 h per day (46.7%). When comparing the sample by nationalities, significant differences were found between some of the measured demographic variables, such as parental marital status, living situation and use of social media platforms. More specifically, results regarding parental marital status showed significant differences for different levels: Spanish participants reported higher rates of married parents (71.3% vs. 60.0%; χ^2^ (1, *n* = 1732) = 18.00, *p* = 0.000) and widowed parents (4.4% vs. 2.1%; χ^2^ (1, *n* = 1732) = 4.47, *p* = 0.0035) whilst American participants had more divorced parents than Spanish students (30.5% vs. 22.5%; χ^2^ (1, *n* = 1732) = 10.54, *p* = 0.001), and reported more frequently other parental marital situations (7.4% vs. 1.8%; χ^2^ (1, *n* = 1732) = 32.49, *p* = 0.000).

Furthermore, regarding the living situation of participants, 62.4% of Spanish students lived with their parents, in comparison to 10.40% of the American students (χ^2^ (1, *n* = 1656) = 363.55, *p* = 0.000). American participants reported living in student apartments more than Spanish students (χ^2^ (1, *n* = 1656) = 19.14, *p* = 0.000), and it was also more frequent for American students to live in on campus residences (χ^2^ (1, *n* = 1656) = 525.21, *p* = 0.000) and to live alone (χ^2^ (1, *n* = 1656) = 21.52, *p* = 0.000). Finally, results regarding use of social media platforms showed that Spanish students reported using Instagram with a higher frequency (χ^2^ (1, *n* = 1772)= 56.44, *p* = 0.000), and WhatsApp with a higher frequency (χ^2^ (1, *n* = 1775)= 647.47, *p* = 0.000) than American students, whilst American participants reported using Twitter with a higher frequency (χ^2^ (1, *n* = 1743) = 77.00, *p* = 0.000) and YouTube with a higher frequency (χ^2^ (1, *n* = 1769) = 17.51, *p* = 0.000), more often than Spanish participants. 

### 3.2. Prevalence of Sexting Behaviors

The comparative prevalence rates of sexting behaviors by nationality, for the total sample are shown in Table 2. For the primary sexting, prevalence rates were higher for the American students (55.0%) than for the Spanish students (35.8%). Results showed that American students were 2.19 times more likely to create and send their own sexual content than Spanish participants. For the secondary sexting, Spanish students were 96% more likely to receive sexts than American students (57.6% vs. 40.9%) American students were 5.52 times more likely to be victims of nonconsensual dissemination of their sexual content (15.0% vs. 3.1%), than Spanish participants. 

With regards to sex and nationality differences, the comparative prevalence rates are shown in Table 3. For primary sexting, American men from our sample were 8.92 times more likely to create and send their own sexual content to others than Spanish men from our sample (51.5% vs. 35.1%; *p*= 0.003), and American women from our sample were 2.25 times more likely to create and send their own sexual content to others than Spanish women from our sample (56.1% vs. 36.2%; *p*= 0.000). 

For secondary sexting behaviors, Spanish men reported higher prevalence rates for receiving sexts (63.0%) than American men (44.6%), χ^2^ (1, *n* = 460)= 22.30, *p* = 0.000, Orspain = 2.94, 95% CI [1.86 4.65]; as did Spanish women in comparison to American women (56.7% vs. 42.3%; *p* = 0.000). Our results show that American women from our sample were 7.05 times more likely to be victims of nonconsensual dissemination of their sexual content than Spanish women (18.9% vs. 3.2%; *p* = 0.000). No significant differences were found between American and Spanish males for being a victim of nonconsensual dissemination of sexts.

When comparing male and female participants within nationality samples, for the Spanish sample results only showed significant differences between men and women for secondary sexting (χ^2^ (1, *n* = 1357) = 4.23, *p* = 0.040, OR = 1.30 95% CI [1.01, 1.66]), whilst significant differences between males and females for the American sample were found for being a victim of nonconsensual dissemination of sexting (χ^2^ (1, *n* = 413) = 15.20, *p* = 0.000, OR = 7.62 95% CI [2.33, 24.87]).

### 3.3. Descriptive Statistics for Sexting-Related Characteristics

The descriptive statistics for the different variables measured for all of the studied sexting behavior are shown in Table 4. For the primary sexting, the mean age of first contact with the behavior for the total sample was 17.4 years old (SD = 3.96). In general, for both nationalities, more participants perceived a high risk in the behavior than any of the other lower-risk options (18.3% vs. 16.0% vs. 13.1%). Out of all of the American students who answered affirmatively to engaging in primary sexting, 50.5% had sent their sexual content to their partner, in comparison to 29.7% of the Spanish students. Out of all the American students who created and sent their own sexual content, in 28.6% of the cases the sexual intensity of the content that was sent was clearly sexual, in comparison to the Spanish sample, where the content was clearly sexual in 12.7% of the cases. Finally, almost 70% of American participants who had created and sent their own sexual content did so with flirting motives, in comparison to the 27.8% of Spanish participants. 

For secondary sexting, Spanish students started to receive sexts at a mean age of 17.2 years old, and the American students at a mean age of 15.5 years old. Regarding how upset they felt by receiving the content, most of the participants answered that they felt barely upset (18.7%), in comparison to moderately upset (17.7%) and very upset (13.7%). In most of the cases, the sender of the sexual content was a friend for both the Spanish sample (31.5%) and the American sample (25.8%). Out of the participants who received sexts, almost one third had received clearly sexual images, and the most common motives were “joke” (30.5%) and “flirting” (28.7%) for both nationalities. 

Finally, for being a victim of nonconsensual dissemination of sexting, American participants reported that the age of first contact with the behavior was at a mean age of 12.4 years old, whilst the Spanish participants reported a mean age of 17.1 years old. Out of the American participants, 11.6% had been very upset about the nonconsensual dissemination of their sexual content, in comparison to only 2.3% of the Spanish participants being very upset. In most of the cases, American participants reported that the shared sexual content was mildly sexual (8.0%), in 6.1% of the cases it was clearly sexual, and in 1.7% of the cases it was subtle. For the Spanish participants, all levels of sexual intensity were between 1% and 2%. With regards to the perpetrator of the nonconsensual dissemination of the sexual content, in the American sample, in 16.7% of the cases the perpetrator was the ex-partner. In the Spanish sample, the ex-partner was the perpetrator in 1% of the cases. The second most prevalent distributer for both samples was a friend (Spain = 1.7%; USA = 8.3%). Ultimately, for motive American participants reported that in most cases the nonconsensual dissemination was carried out as a joke (10.7%) followed by coercion (9.8%) and in 7.8% of the cases to bother them. For the Spanish participants, the most prevalent motive was bothering (1.5%).

## 4. Discussion

Despite the growing body of research regarding sexting and online sexual victimization, there is little evidence exploring cultural differences in association with those behaviors [28,29]. The aim of this study was to examine cultural differences in sexting practices by comparing an American sample and a Spanish sample of university students, and we hypothesized that American and Spanish university students would engage differently in all of the measured sexting behaviors, due to the disparate historical, cultural, political, and socioeconomic factors between countries. 

Prevalence rates reported in this study are consistent with both Spanish and American previously reported sexting prevalence rates [4,8,9,10], but results regarding cross-cultural differences have not been previously analyzed. 

According to our results, although American students started using the internet at an earlier age, internet use and integration of technology into daily lives in Spain are similar to the United States, so, if sexting has to do with opportunity, similar rates should be expected. Nevertheless, overall a clear difference in sexting behaviors between the two countries was found, with Americans being more likely to send sexts and showing more victimization by the nonconsensual dissemination of sexts than Spaniards, and with Spaniards being more likely to receive sexts than Americans, which would support our main hypothesis. It has been previously reported that those who engage more frequently in primary sexting are more likely to become victims of nonconsensual dissemination of sexting, which would explain the higher victimization rates reported by American students [4]. However, Spaniards reported receiving more sexts than Americans. These results could be explained by the accessibility of technology and globalization of sexting [30], since Spanish sexters could be sending sexts to a broader number of recipients or participants could be receiving sexts from people who are not Spanish, thus explaining why they sext less but receive more sexts. Furthermore, active sexting could be underreported in comparison with passive sexting, with an underreporting bias in Spanish population but not in Americans. 

A simple permissive- nonpermissive dichotomy is not enough to account for variations across countries, and a more pluralistic landscape of sexual values should be taken into account. Traditionally, Americans were less tolerant of premarital and teenage sex than other nations and were included in the Sexual Conservatives’ cluster or nations, whilst Spain had a moderate profile similar to the average [31]. On the other hand, it has been stated that Spain progressively moves from a sexual culture in which fear predominated and was based on Catholic morality to a so-called “eroticization of everyday life”, yet Spanish “sexual revolution” has not been as pronounced as in other European countries because of the weight of the Catholic cultural tradition [32]. Nowadays, in Spain more traditional conceptions and behaviors towards sexuality coexist with other more heterodox ones based on different sociodemographic features, but normative sexuality models continue to be valid [33].

Nevertheless, the normalization of intimate behaviors occurring through technology and risk exposure may make the difference regarding sexting, as it has been suggested that the integration of technology into our lives can impact the perceived acceptability of behaviors as well as observance of transgression. Accordingly, it has been reported that Americans show higher scores for normalization of intimate behaviors though technology and higher risk exposure [34].

Additionally, nation-based populations are becoming increasingly diverse, especially in the United States and attitudes towards sexuality vary widely across diverse ethnic groups. Yet, acculturation to mainstream American culture has been associated with more liberal attitudes towards sexuality [35]. It should be noted that since the American sample used was collected from Texas State University, results might not be representative of the American Culture, but rather of a small subgroup of Americans. According to the US Census Bureau in 2018, Hispanics comprised 53.6% of the Texas population, and, thus, it would be possible that our American sample was composed of a high number of Hispanic and Latino participants. Engagement in sexting practices have been previously compared between Colombia and Spain by Gil-Lario et al. [22]. Their recent cross-cultural study results indicate a higher sexting engagement in Colombian adolescents, with authors suggesting that cultures with greater gender inequality cultural values present greater sexual behaviors.

Demographic differences between samples could also be related with differences in sexting frequencies. Spanish students had a higher percentage of married parents, were more likely living with their parents and less likely working partial time. Parental divorce has been linked to some risky sexual behaviors, and perceiving high conflict in parents’ marriages has also been related with more sexual activity and engaging in more risk practices [36]. Lifestyle may further condition sexting involvement. Routine activities theory draws attention to structural opportunities that converge in time and space [37]. The college campus, living at student apartments or living alone was more frequent among American students, which could provide a prone environment to sexual exploration [34]. Working part time and living alone could influence sexting practices according to the routine activity theory, as more free time and independence may increase unsupervised time and the absence of informal control [37].

Risk perception has been associated with sexting engagement, which could also explain why American participants engage more in sexting than Spanish participants [14,17]. Accordingly, a higher percentage of Spanish students thought that engaging in primary sexting posed a high risk, whilst a higher percentage of American students thought that engaging in this behavior posed a moderate risk. These results are in line with previous research, which found that those who perceive a higher risk in sexting are expected to sext less [17]. Furthermore, the intensity of the shared sexual content was overall higher for the American sample than for the Spanish sample, again supporting the role of risk perception in sexting. 

In addition to a higher risk perception of primary sexting, Spanish students felt much more upset about the received sexual content than Americans did, and the content they received was categorized more frequently as clearly sexual. In Spain, it has been previously reported that participants frequently felt upset when engaging in sexting behaviors [6]. Upsetness with receiving sexual content might also be in line with the hypothesis that Spaniards might be more sexually traditional, and might be less used to being exposed to sexual content, thus feeling more distress when they receive it and overstating the intensity of sexual material. On the other hand, in light of our results of a lower frequency of sending sexts and victimization and a higher frequency of receiving sexts in Spaniards, differences in sexual content may be related to a different pattern of sexting, in which the content sent is of low intensity and the content received might not be self-generated by the sender and therefore closer to pornographic sexual content.

Finally, differences were also found for characteristics related to being a victim of nonconsensual dissemination of sexting. American students were victims at a much earlier age than Spanish students (12 vs. 17 years old), and were much more upset about the victimization than Spaniards. Furthermore, and in line with the rest of our results, the American students’ disseminated content involved in victimization was of greater sexual intensity, and they reported more frequently that the dissemination happened because of coercion or as a joke. Motive for sexting could also be attenuating the intensity of the content and the impact on the victim, as sexting related victimization behaviors because of boredom could possibly be less likely to involve clearly sexual content and less likely to cause distress.

When examining results by sex, significant differences between both countries remain, with American males and females engaging more frequently in primary sexting and being more victims of NCDS than Spanish participants, and with Spanish males and females receiving more sexts than American participants. Additionally, Spanish males were more likely to receive sexts than Spanish females, and American females were almost eight times more likely to be a victim of nonconsensual dissemination of sexts than American males. These findings are consistent with previous research which states that women are more frequently involved in passive sexting and more likely to be sexually victimized online than men [38,39]. Although our results show differences between the Spanish and the American samples that might be modulated by cultural factors, the vulnerability of females regarding sexting victimization remains unchanged. Furthermore, according to our results, cultural influence may be more intense in females. For example, American females were around seven times more likely to be a victim of nonconsensual dissemination of sexts than Spanish females, whilst no differences were found for males. American males were almost two times more likely to sext than Spanish males. Baumeister [40] already suggested that females’ sexual behaviors tend to be more variable, flexible, and subject to social and cultural influences than males’, whereas males’ sexual behaviors tend to be more rigid, inflexible, and channeled by biological urges than females’. 

Our results provide new data regarding sexting behavior cross-cultural differences, yet our study has several limitations that should be considered when interpreting the results. First, the sample used was nonprobabilistic and relied on self-report data, and the sample was composed of university students, rather than the general population, so generalization of results should be cautiously done. In this sense, the sample used was self-selected using an online survey, which would explain why the total sample is unbalanced by gender and by nation, which might affect the reliability of results. Furthermore, it should be noted that the Spanish sample was collected using university students from all over Spain, but the American sample is only of Texas State University students, which could affect the reliability of research conclusions. Finally, this study is a cross-sectional investigation, and not longitudinal, so no temporal relationships can be established between the examined variables and sexting behaviors.

## 5. Conclusions

In conclusion, this paper contributes to the first studies examining cultural differences in sexting practices by comparing a Spanish and an American sample of university students. We hypothesized that American and Spanish university students would engage differently in all of the measured sexting behaviors, and our results confirm our hypothesis. Overall, significant differences were found between Americans and Spaniards in all of the measured sexting practices prevalence, and, additionally, significant differences between both samples were found in the measured sexting-related issues such as perceived risk, upsetness, sender and receiver of the sext, intensity of the sexual content and motive for sexting, indicating that there might be culturally-specific pathways to sexting. Finally, sex differences in sexting practices might be modulated by culture, since differences from the total samples were maintained when analyzing results by sex. Yet, our results confirm a higher vulnerability of females regarding sexting victimization in both cultures. These overall results contribute to a deeper understanding of sexting dynamics in young adult population across-countries. These findings can be useful and should be taken into consideration when designing prevention and intervention strategies, and prevention campaigns should be culturally conditioned. Further research should also explore the reasons for these cultural differences, and what social factors might be influencing cultural differences in sexting practices.

## Figures and Tables

**Table 1:** Descriptive statistics for demographic and background variables.

	% (N= 1799)	Mean (SD)	Spain % (N = 1386)	USA % (N = 413)
**Sex**					
Male	26.00		26.40	24.50
Female	74.00		73.60	75.50
**Age**		21.26 (4.61)	21.43 (4.86)	20.70 (3.64)
**Marital Status**					
Single	55.00		54.60	56.20
In relationship	41.70		42.00	40.90
Married	1.50		1.20	2.40
Common law partner	1.10		1.30	0.20
Divorced/separated	0.70		0.90	0.20
**Parental Marital Status**					
Married	68.80		71.30	60.00
Divorced/separated	24.30		22.50	30.50
Widow	3.90		4.40	2.10
Other	3.10		1.80	7.40
**Academic Situation**				
Undergraduate	93.10		92.40	95.40
Master’s degree	3.30		4.00	1.20
Erasmus	1.20		1.50	0.50
International student	0		0	0
Other	2.40		2.20	2.90
**Living Situation**				
With parents	50.2		62.40	10.40
Student apartment	24.8		22.40	32.70
Off Campus student residence	4.10		4.60	2.40
On Campus student residence	9.50		0.70	38.30
Alone	5.10		3.80	9.40
With partner	6.30		6.20	6.80
**Employment Status**				
Unemployed	63.20		67.40	49.40
Employed full time	5.30		5.10	6.10
Employed partial time	31.40		27.40	44.60
**Own smartphone**	98.30		98.0	99.30
**Age of first phone**		13.62 (3.28)	13.86 (3.42)	12.83 (2.63)
**Age of first internet access**		11.69 (3.69)	12.01 (3.83)	10.67 (2.95)
**Internet Access**				
Mobile phone	88.8		89.80	85.70
Laptop	22.9		27.80	6.50
Desktop PC	5.70		6.00	4.60
Tablet	32.9		30.90	39.50
PlayStation	5.3		5.70	4.10
**Frequency Internet Access**				
Once a week	0.20		0.10	0.50
2–3 times a week	0.60		0.40	1.20
Everyday	33.50		33.0	35.10
2–3h per day	17.60		16.7	20.60
More than 3h per day	46.70		48.0	42.60
**Social Media Use**					
Yes	97.00		97.80	94.20
No	2.60		1.60	5.80
**Frequency of Social Media Use**					
Instagram	87.00		86.90	87.4
Facebook	69.60		69.30	70.7
WhatsApp	78.70		98.10	13.60
Twitter	51.30		47.70	63.40
YouTube	94.40		94.60	93.7

**Table 2:** Prevalence of sexting behaviors for the total sample by nationality.

	Spain		USA		
	N	Prev %	N	Prev %	Sig. Test, OR
Primary sexting	1386	35.8	413	55.0	χ^2^ (1, *n* = 1799) = 48.68, *p* = 0.000, OR = 2.19, 95% CI [1.75, 2.74]
Secondary sexting	1386	57.6	413	40.9	χ^2^ (1, *n* = 1799) = 35.68, *p* = 0.000, OR Spain = 1.96, 95% CI [1.57, 2.45]
Being a victim of nonconsensual dissemination	1386	3.1	413	15.0	χ^2^ (1, *n* = 1799) = 82.12, *p* = 0.000, OR = 5.52, 95% CI [3.68, 8.28]

**Table 3:** Prevalence of sexting behaviors by sex and nationality.

**Men (N = 460)**					
	Spanish		USA		
	N	Prev %	N	Prev %	Significance Test, OR
Primary sexting	359	35.1	101	51.5	χ^2^ (1, *n* = 460) = 8.92, *p* = 0.003, OR= 1.96, 95% CI [1.26, 3.07]
Secondary sexting	359	63.0	101	44.6	χ^2^ 2(1, *n* = 460) = 22.30, *p* = 0.000, Orspain = 2.94, 95% CI [1.86 4.65]
Being a victim of nonconsensual dissemination	359	3.1	101	3.0	χ^2^ (1, *n* = 460) = 0.002, *p* = 0.961, OR = 0.97, 95% CI [.27, 3.54]
**Women (N = 1311)**					
	Spanish		USA		
	N	Prev %	N	Prev %	Sig. Test, OR
Creating and sending nude or sexual imagery of oneself	999	36.2	312	56.1	χ^2^ (1, *n* = 1311) = 38.75, *p* = 0.000, OR = 2.25, 95% CI [1.74, 2.91]
Receiving sexts	998	56.7	312	42.3	χ^2^ (1, *n* = 1310) = 19.82, *p* = 0.000, Or Spain = 1.79, 95% CI [1.38, 2.31]
Being a victim of nonconsensual dissemination	999	3.2	312	18.9	χ^2^ (1, *n* = 1311) = 90.81, *p* = 0.000, OR = 7.05 95% CI [4.48, 11.07]

**Table 4:** Descriptive statistics for different sexting variables and characteristics.

	Total Sample % (N = 1799)	Spain % (N = 1386)	USA % (N = 413)	Significance Test
**Primary sexting**				
Age of first contact	17.43 (3.96)	17.89 (3.81)	16.45 (4.11)	T (758) = 4.72, *p* = 0.000, d = 0.37, 95% CI [.22, 0.52]
Perceived risk				
*Low risk*	13.10	10.50	21.50	χ^2^ (1, *n* = 1799) = 34.00, *p* = 0.000, OR = 2.33, 95% CI [1.75, 3.12]
*Moderate risk*	16.00	14.20	22.00	χ^2^(1, *n* = 1799) = 14.47, *p* = 0.000, OR = 1.71, 95% CI [1.29, 2.25]
*High risk*	18.30	19.30	15.30	χ^2^(1, *n* = 1799) = 3.42, *p* = 0.065, OR = 0.75, 95% CI [0.56, 1.02]
Receiver				
*Friend*	3.70	3.20	5.30	χ^2^(1, *n* = 1799) = 3.84, *p* = 0.050, OR = 1.68, 95% CI [0.99, 2.83]
*Partner*	25.10	21.80	36.30	χ^2^(1, *n* = 1799) = 35.71, *p* = 0.000, OR = 2.05, 95% CI [1.61, 2.60]
*Ex-partner*	7.60	6.60	10.70	χ^2^(1, *n* = 1799) = 7.34, *p* = 0.007, OR = 1.68, 95% CI [1.15, 2.45]
*Stranger*	0.50	0.30	0.70	χ^2^(1, *n* = 1799) = 1.57, *p* = 0.210, OR = 2.53, 95% CI [0.56, 11.34]
Intensity of sexual content				
*Subtle*	10.50	10.60	10.20	χ^2^(1, *n* = 1799) = 0.065, *p* = 0.800, OR = 0.95, 95% CI [0.66, 1.37]
*Mildly sexual*	14.80	13.60	18.60	χ^2^(1, *n* = 1799) = 6.33, *p* = 0.012, OR = 1.45, 95% CI [1.09, 1.94]
*Clearly sexual*	16.30	12.70	28.60	χ^2^(1, *n* = 1799) = 58.63, *p* = 0.000, OR = 2.75, 95% CI [2.11, 3.59]
Motive				
*Flirting*	27.40	20.70	49.20	χ^2^(1, *n* = 1769) = 128.75, *p* = 0.000, OR = 3.70, 95% CI [2.93, 4.67]
*Joke*	10.10	12.20	2.90	χ^2^(1, *n* = 1769) = 30.49, *p* = 0.000, OR Spain = 4.66, 95% CI [2.57, 8.47]
*Bothering*	0			-
*Coercion or threats*	1.90	1.30	3.90	χ^2^(1, 1769) = 11.87, *p* = 0.001, OR = 3.17, 95% CI [1.59, 6.34]
**Secondary sexting**				
Age of first contact	16.87 (3.81)	17.20 (3.08)	15.51 (5.75)	T (191.20) = 3.69, *p* = 0.000, d = 0.45, 95% CI [0.27, 0.62]
Upsetness				
*Barely upset*	18.70	20.10	14.30	χ^2^(1, *n* = 1799) = 6.96, *p* = 0.008, OR Spain = 1.51, 95% CI [1.11, 2.04]
*Moderately upset*	17.70	18.50	14.80	χ^2^(1, *n* = 1799) = 3.11, *p* = 0.078, OR= 0.76, 95% CI [0.56, 1.03]
*Very upset*	13.70	14.90	9.70	χ^2^(1, *n* = 1799) = 7.23, *p* = 0.007, OR Spain = 1.63, 95% CI [1.14, 2.33]
Sender				
*Friend*	21.10	22.70	16.00	χ^2^(1, *n* = 1799) = 8.51, *p* = 0.004, OR Spain = 1.54, 95% CI [1.15, 2.06]
*Partner*	2.80	3.30	1.20	χ^2^(1, *n* = 1799) = 5.13, *p* = 0.023, OR Spain = 2.80, 95% CI [1.11, 7.10]
*Ex-partner*	2.60	2.20	3.60	χ^2^(1, *n* = 1799) = 2.49, *p* = 0.115, OR= 1.65, 95% CI [0.88, 3.08]
*Stranger*	12.30	13.30	9.00	χ^2^(1, *n* = 1799) = 5.67, *p* = 0.017, OR Spain = 1.57, 95% CI [1.08, 2.27]
Intensity of sexual content				
*Subtle*	5.70	6.30	3.60	χ^2^(1, *n* = 1799) = 4.16, *p* = 0.041, OR Spain = 1.74, 95% CI [1.00, 3.04]
*Mildly sexual*	14.10	15.70	9.00	χ^2^(1, *n* = 1799) = 11.77, *p* = 0.001, OR Spain = 1.89, 95% CI [1.31, 2.72]
*Clearly sexual*	29.80	31.00	25.90	χ^2^(1, *n* = 1799) = 3.98, *p* = 0.046, OR Spain = 1.29, 95% CI [1.00, 1.65]
Motive				
*Flirting*	19.90	21.00	16.20	χ^2^(1, *n* = 1772) = 4.62, *p* = 0.032, OR Spain = 1.38, 95% CI [1.03, 1.84]
*Joke*	21.20	24.90	9.00	χ^2^(1, *n* = 1772) = 48.07, *p* =0.000, OR Spain = 3.36, 95% CI [2.35, 4.82]
*Bothering*	4.10	4.00	7.70	χ^2^(1, *n* = 1772) = 17.95, *p* = 0.000, OR= 2.83, 95% CI [1.76, 4.55]
*Coercion*	1.30	.40	4.40	χ^2^(1, *n* = 1772) = 39.37, *p* = 0.000, OR = 12.34, 95% CI [4.55, 33.45]
**Being victim of nonconsensual dissemination**				
Age of first contact	13.94 (8.23)	17.09 (4.79)	12.40 (9.09)	T (128.22) = 3.87, *p* = 0.000, d = 0.59, 95% CI [0.22, 0.96]
Upsetness				
*Barely upset*	1.90	2.00	1.50	χ^2^(1, *n* = 1799) = 0.552, *p* = 0.457, OR = 0.72, 95% CI [0.29, 1.74]
*Moderately upset*	1.00	0.02	3.60	χ^2^(1, *n* = 1799) = 37.47, *p* = 0.000, OR = 10.43, 95% CI [3.77, 28.86]
*Very upset*	4.40	2.30	11.60	χ^2^(1, *n* = 1799) = 64.95, *p* = 0.000, OR= 5.56, 95% CI [3.51, 8.83]
Sender				
*Friend*	1.90	1.20	4.10	χ^2^(1, *n* = 1799) = 14.33, *p* = 0.000, OR = 3.46, 95% CI [1.75, 6.83]
*Partner*	0.60	0.40	1.50	χ^2^(1, *n* = 1799) = 6.24, *p* = 0.012, OR= 4.07, 95% CI [1.24, 13.41]
*Ex-partner*	2.30	0.60	8.20	χ^2^(1, *n* = 1799) = 81.78, *p* = 0.000, OR = 15.45, 95% CI [7.09, 33.66]
*Stranger*	0.30	0.30	0.50	χ^2^(1, *n* = 1799) 0.366, *p* = 0.545, OR = 1.68, 95% CI [0.31, 9.21]
Intensity of sexual content				
*Subtle*	1.70	1.70	1.70	χ^2^(1, *n* = 1799) = 0.003, *p* = 0.960, OR = 0.98, 95% CI [0.42, 2.29]
*Mildly sexual*	2.60	1.00	8.00	χ^2^(1, *n* = 1799) = 60.93, *p* = 0.000, OR = 8.51, 95% CI [4.51, 16.07]
*Clearly sexual*	2.20	1.10	6.10	χ^2^(1, *n* = 1799) = 36.17, *p* = 0.000, OR = 5.89, 95% CI [3.08, 11.28]
Motive				
*Flirting*	.50	0.40	1.00	χ^2^(1, *n* = 1797) = 2.35, *p* = 0.125, OR = 2.70, 95% CI [0.72, 10.09]
*Joke*	1.80	0.70	5.30	χ^2^(1, *n* = 1797) = 38.55, *p* = 0.000, OR= 7.73, 95% CI [3.63, 16.46]
*Bothering*	1.60	0.90	3.90	χ^2^(1, *n* = 1797) = 18.75, *p* = 0.000, OR = 4.61, 95% CI [2.16, 9.82]
*Coercion*	1.60	0.60	4.80	χ^2^(1, *n* = 1797) = 37.71, *p* = 0.000, OR = 8.75, 95% CI [3.83, 20.03]

## Data Availability

Not applicable.
